# Editorial: Diets and eating patterns: effects on the immune system and its regulation

**DOI:** 10.3389/fnut.2023.1305736

**Published:** 2023-11-09

**Authors:** Cristina Torres-Fuentes, Raphael Chevre, Almudena Ortega-Gomez

**Affiliations:** ^1^Nutrigenomics Research Group, Department of Biochemistry and Biotechnology, Universitat Rovira i Virgili, Tarragona, Spain; ^2^Institute of Experimental Pathology (ExPat), Center of Molecular Biology of Inflammation (ZMBE), University of Münster, Münster, Germany; ^3^Department of Endocrinology and Nutrition, Virgen de la Victoria University Hospital, Málaga, Spain; ^4^Biomedical Research Institute of Malaga and Platform in Nanomedicine (IBIMA-BIONAND Platform), Málaga, Spain; ^5^Centro de Investigación Biomédica en Red in Physiopathology of Obesity and Nutrition (CIBEROBN), Instituto de Salud Carlos III, Madrid, Spain

**Keywords:** diet, immune system, inflammation, time-restricted eating, dietary inflammation index (DII), leukocyte

Diets and eating patterns have far-reaching impacts on our physical health and wellbeing. Food intake interferes with a wide variety of physiological processes, including changes in leukocyte levels, hyperlipidemia, and release of pro- or anti-inflammatory factors, among others. Therefore, understanding the modulation of the immune system by diet is essential to maintain physiological homeostasis.

Time-restricted eating, a form of dietary restriction based on intermittent fasting supporting circadian rhythms, has emerged as a common eating pattern (1). Previous studies have demonstrated that time-restricted eating confers metabolic benefits, such as reduction of body weight, adiposity, and insulin resistance (2). In this Research Topic, Song et al. investigated whether time-restricted feeding (TRF) can improve inflammatory bowel disease (IBD) in a mouse model. The development of the disease was studied in groups of mice that followed a fasting-mimicking diet (FMD) or a TRF pattern (6h feeding during the dark phase). The TRF and FMD groups showed a significant decrease in the disease scores and reduced systemic and intestinal inflammation. In addition, both groups increased the number of colonic crypts and reduced histological scores, the percentage of peripheral and mesenteric lymph node CD4^+^ cells, infiltration of leukocytes, and the presence of macrophages at the crypt base of the colon. However, the TRF group failed at promoting regeneration and repair of the intestinal epithelium, as opposed to the FMD group, which could be the reason behind the moderately better effect of IBD development of FMD over TRF patterns.

In animal obesity models, myelopoiesis is expanded in the bone marrow, resulting in increased numbers of circulating monocytes and adipose tissue macrophages (3, 4). Kim et al. studied the effect of TRF in the hematopoietic stem and progenitor cell niche (HSPC) in a murine obesity model. After 6 weeks of high-fat diet (HFD), mice could access HFD *ad libitum* or on a 10 h TRF pattern. These mice were compared to a control group with low-fat diet (LFD) *ad libitum*. The authors observed that mice following TRF presented reduced numbers of monocytes and neutrophils in the bone marrow and circulation. Interestingly, B, T, and natural killer (NK) cell circulation levels were unaffected. Among the network of transcription factors that regulate the hematopoietic lineages, only *Cebpa* presented elevated mRNA expression levels after HFD *ad libitum*, which TRF normalized. Consistent with this finding, the elevations in myeloid progenitor populations inflicted by HFD *ad libitum* were restored by TRF intervention. Since normoglycemia was restored to LFD levels in the TRF groups and increased circulating monocytes and neutrophils, as well as myelopoiesis (but no lymphocytes), have been previously observed in diabetic mice (5), Kim et al. argued that the protective role of TRF may be in part mediated through lowering HDF-induced hyperglycemia. These findings support the notion that following circadian rhythms in feed and fast cycles improves cardiometabolic values and immune regulation of inflammatory diseases, such as IBD and obesity.

Over the past few decades, the occurrence of obesity and allergies has risen, and several clinical studies have attempted to establish a relationship between these two conditions (6). Recently, it has been discovered that obesity is linked to high levels of serum IgE (7). Avila Castillo et al. hypothesized that circulating 25(OH)D levels are negatively related to circulating allergen-specific IgE. They used a population-based cohort study with a baseline examination of 10.0000 randomly selected participants from Leipzig (Germany). The visceral and subcutaneous adipose tissue (VAT and SAT) of a sub-cohort of 1,032 participants was estimated using MRI, and circulating levels of IgE and 25(OH)D were measured. They found that participants with higher BMI presented lower levels of 25(OH)D, as previously reported. In that respect, a strong intercorrelation between vitamin D receptor (VDR) gene expression in SAT and VAT and circulating 25(OH)D was found. However, no connection was found between circulating IgE and BMI or fat distribution. Thus, more research is needed to fully understand the molecular mechanisms linking allergy and obesity.

Proposed in 2014, the Dietary Inflammatory Index (DII) has emerged as a valuable tool to control diet-modulated inflammation (8). In this Research Topic, Zhou et al. investigated the association between the Dietary Inflammatory Index (DII) and hypertension, a common clinical syndrome where inflammation plays a pivotal role. In this weighted cross-sectional study based on the NHANES, 45.023 participants were included, representing 191 million adults in the United States. Interestingly, hypertensive participants presented higher values of DII compared to non-hypertense participants, and as expected, the Healthy Eating Index (HEI) correlated negatively with DII. In particular, dietary fiber, vitamin A, beta-carotene, niacin, and caffeine were the most strongly associated variables with hypertension. Furthermore, the authors developed a nomogram model based on key dietary factors to identify hypertension risk. This model showed favorable discriminatory power, constituting a promising prevention tool to screen the risk of developing hypertension.

A suboptimal dietary intake is particularly problematic for immunocompromised patients since it increases their risk of suffering metabolic comorbidities. This is the case of patients with HIV (67% of whom live in Sub-Saharan Africa). Antiretroviral therapy on these patients reports limited benefits when they follow a suboptimal diet. Kiyimba et al. conducted a cross-sectional study among people living with HIV (PLWH), assessing their dietary and energy intake and analyzing circulating cardiometabolic factors. They found that Ugandan PLWH have a high prevalence of low HDL-c, abdominal obesity, raised fasting blood glucose, hypertension, and elevated triglycerides, which translate into a high prevalence of metabolic syndrome. These parameters can be partially explained by the predominantly carbohydrate-based food consumed by the cohort, with minimal intake of fruits and vegetables. Consequently, a high percentage of participants showed limited intake of Zn and Ca and B vitamins, which is related to inadequate clinical outcomes of HIV treatment (9).

In summary, this Research Topic emphasizes the significance of immunonutrition in mitigating inflammatory diseases by providing new insights into the impact of nutrition and dietary patterns on inflammatory and immune conditions.

## Author contributions

CT-F: Writing—review & editing. RC: Writing—review & editing. AO-G: Writing—original draft, Writing—review & editing.
